# Association between maternal anti-Ro and anti-La antibody levels and congenital heart block: a 20-year cohort study

**DOI:** 10.1007/s00404-026-08360-z

**Published:** 2026-02-21

**Authors:** Gabriel Duque Pannain, Joelma Queiroz Andrade, Marco Antônio Borges Lopes, Fabrício Marcondes Camargo, Vera Lúcia Jornada Krebs, Werther Brunow de Carvalho, Rossana Pulcinelli Vieira Francisco

**Affiliations:** 1 Division of Fetal Medicine, Department of Obstetrics, School of Medicine, University of São Paulo (FMUSP), São Paulo, São Paulo Brazil; 2 Division of Neonatology, Department of Pediatrics, School of Medicine, University of São Paulo (FMUSP), São Paulo, São Paulo Brazil

**Keywords:** Pregnancy, Anti-Ro antibodies, Anti-La antibodies, Congenital heart block, Lupus erythematosus, Sjögren’s syndrome

## Abstract

**Objective:**

To evaluate the association between maternal anti-Ro and anti-La antibody levels and the occurrence of congenital heart block (CHB) in fetuses and newborns.

**Methods:**

This retrospective cohort study included 182 pregnant women with positive anti-Ro and/or anti-La antibodies who received prenatal care at our tertiary center between 2002 and 2022. Maternal clinical, laboratory, and obstetric variables were analyzed.

**Results:**

Thirteen fetuses (7.1%) were diagnosed with CHB. Mothers of affected fetuses had significantly higher anti-Ro (median 240 vs. 42; *p* < 0.001) and anti-La (median 150 vs. 10; *p* < 0.001) levels. Anti-La positivity was more frequent in the CHB group (76.9% vs. 42.6%; *p* = 0.017). Lower complement C4 levels (*p* = 0.008) and disease duration of less than 1 year since diagnosis (*p* = 0.008) were also associated with CHB. Preconception hydroxychloroquine and prednisone use were less frequent in affected pregnancies (*p* = 0.044 and *p* = 0.039, respectively).

**Conclusion:**

Higher maternal anti-Ro and anti-La antibody levels were significantly associated with fetal CHB. Preconception hydroxychloroquine may provide a protective effect. Early diagnosis and specialized care are essential for optimizing neonatal outcomes.

## Introduction

During pregnancy, a state of physiological immunomodulation occurs. It is known that there is a decrease in cytokines related to T helper 1 (Th1) lymphocytes, an increase in the suppressive response of T helper 2 (Th2) lymphocytes, and suppression of Natural Killer (NK) cell activity [1, 2]. These alterations appear to explain why diseases such as rheumatoid arthritis (RA) often improve during pregnancy, while others, such as systemic lupus erythematosus (SLE), tend to worsen during this period [3].

SLE is a chronic inflammatory disease characterized by the production of autoantibodies directed against auto-antigens, resulting in immune dysregulation that primarily affects young female patients [4]. Given this epidemiological profile, it is not uncommon for SLE to be associated with the gestational and puerperal periods, potentially contributing to the development of pregnancy-related complications [5].

Similar to other rheumatologic diseases, such as antiphospholipid antibody syndrome and Sjögren’s syndrome (SS), SLE is associated with class IgG autoantibodies, which are known to cross the placental barrier, unlike class IgM autoantibodies [6]. This transplacental passage occurs via pinocytosis in the syncytiotrophoblast, where the antibodies bind to fetal receptors and are transported to the trophoblastic basal lamina. Consequently, they gain access to the endothelium and fetal circulation, potentially causing severe and irreversible fetal injury [7].

Among these, two specific IgG autoantibodies—anti-Ro/SS-A and anti-La/SS-B—are of particular concern during pregnancy. They are more prevalent in patients with SS, SLE, and RA [8]. In these patients, such antibodies appear to be associated with unexplained pregnancy loss and adverse obstetric outcomes, as well as neonatal lupus syndrome and congenital heart block, conditions in which the fetus presents with a ventricular rate lower than expected, potentially progressing to significant cardiac failure or even intrauterine fetal demise [9, 10].

Congenital heart block is the most severe and clinically relevant manifestation of cardiac neonatal lupus, occurring in approximately 1–2% of pregnancies exposed to maternal anti-Ro/SSA and/or anti-La/SSB antibodies, with a recurrence rate reaching 15–20% in subsequent pregnancies [11–13]. Established risk factors for congenital heart block include high maternal antibody titers, a previous affected fetus, specific antibody profiles (particularly anti-Ro52), and genetic and inflammatory susceptibility of the fetus [14–17]. Increasing evidence suggests that the risk of fetal cardiac injury is not merely associated with antibody positivity, but rather with quantitative antibody levels, highlighting the importance of antibody burden as a determinant of pathogenicity. Several observational studies have demonstrated a dose–response relationship between higher maternal anti-Ro/SSA and anti-La/SSB concentrations and the development of fetal conduction abnormalities, myocardial inflammation, endocardial fibroelastosis, and cardiomyopathy [16].

The pathogenic mechanism underlying fetal cardiac involvement is mediated by active transplacental transfer of maternal IgG antibodies through the neonatal Fc receptor (FcRn), a process that intensifies from the late first trimester onward, coinciding with the vulnerable window for congenital heart block development between 18 and 24 weeks of gestation [6]. Once in the fetal circulation, anti-Ro/SSA and anti-La/SSB antibodies bind to apoptotic cardiomyocytes, triggering inflammatory cascades, macrophage activation, and subsequent fibrosis of the atrioventricular node and surrounding myocardium [11]. Beyond irreversible third-degree congenital heart block, the clinical spectrum of neonatal lupus encompasses a range of cardiac manifestations—including first- and second-degree atrioventricular block, endocardial fibroelastosis, dilated cardiomyopathy, and valvular dysfunction—as well as non-cardiac features such as cutaneous lupus lesions, hepatic involvement, and hematological abnormalities [8–11]. Preventive strategies have therefore focused on immunomodulation, with hydroxychloroquine (HCQ) at a dose of 400 mg daily, initiated before 10 weeks’ gestation, demonstrating a significant reduction in the recurrence of CHB in high-risk pregnancies [13].

Despite these advances, clinically applicable antibody thresholds capable of refining fetal risk stratification remain poorly defined. In this context, the present study aims to investigate the association between quantitative maternal anti-Ro and anti-La antibody levels and the risk of fetal atrioventricular block, seeking to identify antibody profiles that may improve early risk prediction and inform targeted surveillance and preventive strategies.

## Methods

This was a retrospective cohort study including 182 pregnant women who received prenatal care at the Hospital das Clínicas, Faculty of Medicine, University of São Paulo (HCFMUSP), between 2002 and 2022. The study was submitted to the local etics committee and approved under protocol number CAAE 86988425.9.0000.0068.

Because the condition under investigation is relatively rare, a convenience sampling strategy was employed, following the inclusion criteria described above.

### Inclusion and exclusion criteria

**Fig. 1:**
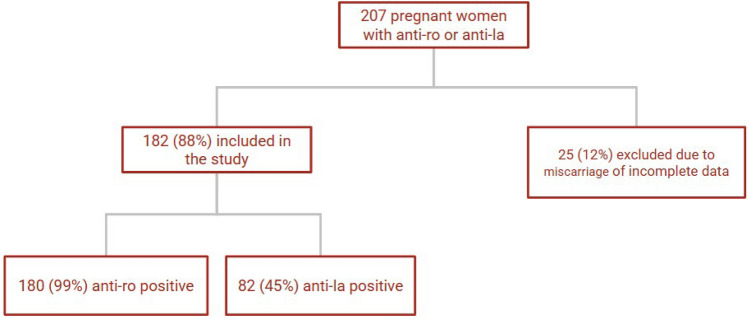
Flowchart with included patients

The inclusion criterion was a singleton pregnancy in patients with positive anti-Ro and/or anti-La antibody titers who underwent both prenatal care and delivery at HCFMUSP between 2002 and 2022. Pregnancies that ended in miscarriage and those with incomplete medical records were excluded from the analysis. Pregnant women with rheumatologic diseases but negative anti-Ro and anti-La antibody titers were excluded from the analysis (Fig. 1).

Although we acknowledge that this is not a well-established protocol, given the known association between anti-Ro and anti-La antibodies and congenital heart block, at our institution, once a diagnosis of congenital heart block is made, anti-Ro and anti-La antibody testing is routinely requested to guide counseling for future pregnancies. Consequently, several patients included in the study did not have a previously diagnosed rheumatologic disease.

For congenital heart block, only fetuses with third-degree atrioventricular block were considered. All cases of congenital heart block were referred to our center from 26 weeks of gestational age onward. None of the cases diagnosed with complete atrioventricular block involved patients who were already receiving prenatal care at our institution.

### Data collection

All variables were entered into a Microsoft Excel spreadsheet and subsequently analyzed using statistical software.

Maternal variables included: maternal age, parity, underlying disease, disease duration, presence of disease activity at conception (presence of disease activity was defined according to the Systemic Lupus Erythematosus Disease Activity Index 2000—SLEDAI-2 K)[14], cutaneous, hepatic, hematologic, renal, and neurologic manifestations, serum levels of anti-Ro and anti-La antibodies [Maternal anti-Ro/SSA and anti-La/SSB antibody levels were quantified using an enzyme-linked immunosorbent assay (ELISA). The same assay methodology was employed consistently throughout the entire study period, ensuring comparability of antibody measurements over time. Given the known variability in antibody titers across different immunoassay platforms, the exclusive use of ELISA minimized inter-assay variability and allowed for reliable longitudinal analysis of quantitative antibody levels across the 20-year cohort], presence of other positive autoantibodies, antinuclear antibody (ANA) pattern and titer, complement levels (C3 and C4) during prenatal follow-up, hydroxychloroquine use (gestational age at initiation and dosage), prednisone use (gestational age at initiation and dosage), and mode of delivery.

Fetal and neonatal variables included: presence of cardiac abnormalities (such as congenital heartblock, second-degree atrioventricular block, endocardial fibroelastosis, cardiomyopathy, or other structural cardiac abnormalities) confirmed by fetal echocardiography, birth status, 5 min Apgar score, need for pacemaker implantation (and timing thereof), and post-implantation complications. Non-cardiac manifestations of neonatal lupus were not evaluated in the present study.

### Statistical analysis

A descriptive analysis of clinical, laboratory, and obstetric variables was initially performed. Continuous variables with normal distribution were expressed as means and standard deviations (SD) and compared between groups (with and without fetal congenital heart block) using the Student’s t-test. Variables with non-normal distribution, as determined by the Shapiro–Wilk and Kolmogorov–Smirnov tests, were expressed as medians and interquartile ranges (IQR), with comparisons made using the Mann–Whitney U test.

Categorical variables were presented as absolute and relative frequencies and compared using the Pearson chi-square test or Fisher’s exact test, as appropriate.

For categorical variables showing a statistically significant association with the outcome, odds ratios (OR) and 95% confidence intervals (95% CI) were estimated using 2 × 2 contingency tables. The significance level was set at 5% (*p* < 0.05).

All analyses were performed using IBM SPSS Statistics for Windows, version 25.0 (Armonk, NY: IBM Corp.).

### Limitations

This study has several methodological limitations that should be acknowledged. Its retrospective design inherently limits control over data collection and is subject to information bias, missing data, and potential misclassification of exposures and outcomes. In addition, the relatively small sample size—particularly with respect to the number of fetuses affected by congenital heart block—may have limited the statistical power to detect modest associations and to perform more granular subgroup or multivariable analyses. The low frequency of congenital heart block, although expected given the rarity of the condition, also restricts the generalizability of the findings. Furthermore, residual confounding cannot be excluded, as unmeasured or incompletely captured clinical variables may have influenced both antibody levels and fetal outcomes. These limitations underscore the need for cautious interpretation of the results and highlight the importance of larger, prospective, multicenter studies to validate the observed associations.

## Results

A total of 182 pregnant women with positive anti-Ro and/or anti-La antibodies were included in the study, of whom 13 (7.1%) had fetuses diagnosed with congenital heart block (CHB). There were no significant differences between the CHB and non-CHB groups regarding maternal age, number of deliveries, or previous miscarriages. For CHB, only fetuses with third-degree atrioventricular block were considered. All cases were referred to our center from 26 weeks of gestational age onward. None of the cases diagnosed with complete atrioventricular block involved patients who were already receiving prenatal care at our institution.

Among the 182 pregnant women, 82 (45%) were positive for anti-La antibodies and 180 (99%) were positive for anti-Ro antibodies. Of the 82 women with anti-La positivity, only 2 had isolated anti-La positivity; all others were positive for both anti-Ro and anti-La antibodies.

When comparing the two groups, pregnant women with affected fetuses had significantly higher median levels of anti-La antibodies (150 vs. 10; *p* < 0.001) and anti-Ro antibodies (240 vs. 42; *p* < 0.001). The frequency of anti-La positivity was also higher among mothers with CHB (76.9% vs. 42.6%; *p* = 0.017). These patients presented with disease duration of less than 1 year since diagnosis (*p* = 0.008) and lower C4 levels (*p* = 0.008).

The presence of rheumatological diseases (including SLE, Sjogren, rheumatoid arthritis, dermatomyositis, mixed connective tissue disease) was more frequent among mothers without affected fetuses (100% vs. 69.2%; *p* < 0.001), as was the presence of isolated SLE (85.8% vs. 38.5%; *p* < 0.001). Conversely, Sjögren’s syndrome was proportionally more common in the CHB group (38.5% vs. 14.2%; *p* = 0.021), as was a history of a previously affected child (50% vs. 7.8%; *p* = 0.014).

Subgroup analyses by disease type (e.g., SLE vs. SS) and treatment exposure did not yield statistically significant results, possibly because the treatment was similar across groups and/or because treatment duration was highly heterogeneous in both groups.

Disease activity at conception (*p* = 0.019), cutaneous involvement (*p* < 0.001), articular manifestations (*p* = 0.021), and positive ANA (*p* = 0.047) were also significantly associated with the outcome.

Pre-pregnancy hydroxychloroquine at a dose of 400 mg daily use was less frequent among mothers with affected fetuses (30.8% vs. 60.5%; *p* = 0.044), as was prednisone use (15.4% vs. 47.7%; *p* = 0.039). Cesarean delivery was more frequent among women with CHB (92.3% vs. 59.9%; *p* = 0.033).

These maternal and fetal characteristics are summarized in Table 1.

**Table 1:** Maternal and fetal characteristics

Variable	CHB – No (*n* = 169)	CHB – Yes (*n* = 13)	*p*-value
Maternal age (years), mean ± SD	29 ± 5	29 ± 7	0.720*
Gravidity, median (IQR)	2 (1–3)	1 (1–2)	0.075**
Parity, median (IQR)	1 (0–1)	0 (0–1)	0.130**
Miscarriages, median (IQR)	0 (0–0)	0 (0–0)	0.549**
Anti-La level, median (IQR)	10 (10–78.5)	150 (97–240)	< 0.001**
Anti-Ro level, median (IQR)	42 (14–200)	240 (179–240)	< 0.001**
Disease duration (years), median (IQR)	5 (2–10)	1 (0–6)	0.008**
C3 level, median (IQR)	113 (96–162)	86 (79–160)	0.093
C4 level, median (IQR)	19 (13–26)	14 (7–15.6)	0.008**
Anti-La positive, *n* (%)	72 (42.6%)	10 (76.9%)	0.017****
SLE diagnosis, *n* (%)	145 (85.8%)	5 (38.5%)	< 0.001****
Sjögren’s syndrome, *n* (%)	24 (14.2%)	5 (38.5%)	0.021****
Disease active at conception, *n* (%)	31 (18.3%)	6 (46.2%)	0.016****
Cutaneous manifestations, *n* (%)	127 (80.9%)	2 (15.4%)	< 0.001***
Articular manifestations, *n* (%)	110 (69.6%)	5 (38.5%)	0.021****
Positive ANA, *n* (%)	132 (78.1%)	7 (53.8%)	0.047****
Pre-pregnancy hydroxychloroquine use at a dose of 400 mg daily, *n* (%)	92 (60.5%)	4 (30.8%)	0.044***
Pre-pregnancy prednisone use, *n* (%)	71 (47.7%)	2 (15.4%)	0.039***
Cesarean delivery, *n* (%)	97 (59.9%)	12 (92.3%)	0.033***

Among all fetuses with CHB (*n* = 13; 100%), the diagnosis was confirmed by neonatal echocardiography. Of these, 62% (*n* = 8) required pacemaker implantation during the neonatal hospitalization. One neonate (7.7%) underwent pacemaker insertion on the first day of life due to a baseline heart rate of 30 bpm and clinical signs of poor peripheral perfusion, whereas the remaining procedures were performed electively during the first week of life.

Post-implantation complications were limited to infection, which occurred in 43% (*n* = 3) of neonates who received pacemakers during hospitalization.

## Discussion

The present study corroborates and extends previous evidence demonstrating that the risk of fetal congenital heart block (CHB) is closely related not only to maternal anti-Ro/SSA and anti-La/SSB antibody positivity, but also to the quantitative burden of these autoantibodies. Seminal studies by Jaeggi et al. and Buyon et al. have consistently shown a direct, dose–response relationship between maternal anti-Ro antibody titers—particularly anti-Ro52 and anti-Ro60—and the incidence of fetal cardiac disease, with higher titers conferring substantially increased risk [15–17]. Jaeggi et al. reported that all cases of cardiac neonatal lupus occurred in mothers with markedly elevated anti-Ro60 levels, frequently exceeding the upper limits of conventional assays, with the risk of fetal atrioventricular block reaching up to 29% in women with anti-Ro60 titers above 50,000 units [15–17]. Similarly, Buyon et al. demonstrated that no cases of CHB occurred in pregnancies with low anti-Ro52 or anti-Ro60 titers, whereas the risk increased progressively across higher antibody quartiles, reaching 7.7% in the highest quartile and up to 27.3% among women with a previously affected child [15–17].

In line with these observations, our findings show that fetuses who developed CHB were exposed to significantly higher maternal antibody levels, with markedly elevated anti-La (median: 150 vs. 10; *p* < 0.001) and anti-Ro titers (median: 240 vs. 42; *p* < 0.001), reinforcing the concept that antibody concentration is a critical determinant of fetal cardiac involvement. Moreover, consistent with prior reports, isolated anti-La/SSB positivity was not independently associated with CHB, but may act as a risk modifier in the presence of anti-Ro/SSA antibodies [15–17]. Finally, in agreement with Kaizer et al., no fetal cardiac involvement was observed in mothers with anti-Ro serum levels below 110, supporting the use of quantitative antibody thresholds as a clinically relevant tool for risk stratification and for guiding intensified fetal surveillance during pregnancy [12].

An important methodological consideration when interpreting antibody thresholds is that anti-Ro/SSA and anti-La/SSB cut-off values are inherently assay-dependent. As antibody titers may vary substantially according to the immunoassay employed, absolute numerical thresholds cannot be universally extrapolated across different laboratory platforms. This limitation has been highlighted in previous studies and must be acknowledged when applying specific cut-off values for clinical risk stratification [15–18]. In the present study, antibody levels were quantified using a single ELISA-based methodology throughout the entire study period, which ensured internal consistency and minimized inter-assay variability. Nevertheless, the cut-off values identified herein should be interpreted within the context of the assay used and may not be directly comparable to thresholds derived from other immunoassays. Despite this limitation, our findings remain clinically relevant, as they reinforce the broader and well-established concept that increasing maternal anti-Ro/SSA antibody burden—rather than mere antibody positivity—is a key determinant of fetal cardiac risk, supporting the use of quantitative antibody assessment to guide fetal surveillance strategies.

Understanding the clinical behavior of these diseases during pregnancy is of fundamental importance [12, 13, 16]. As demonstrated in the present study, in which the mean maternal age was 29 years, connective tissue diseases—although typically diagnosed at advanced ages—can affect women of reproductive age and therefore represent a possible comorbidity in high-risk pregnancies, with important implications during the reproductive period.

Regarding maternal clinical assessment, preconception counseling and the timing of pregnancy are critical considerations. As previously reported, pregestational use of hydroxychloroquine at a dose of 400 mg daily exhibited a protective effect against the development of fetal CHB (60.5% vs. 30.8%; *p* = 0.044) [12, 13, 16]. Conversely, disease activity at conception according to the SLEDAI-2 K criteria was significantly associated with an increased risk of CHB (*p* = 0.016) [14].

An association was also observed between disease duration and the occurrence of CHB, with this complication being more frequent in patients with a disease duration of less than 1 year since diagnosis (*p* = 0.008). This finding may be explained by the fact that patients with longer than 1 year disease since diagnosis are often under regular follow-up and treatment, frequently using preventive medications for congenital heart block, such as hydroxychloroquine. This supports the hypothesis that early and adequate disease management may serve as a protective factor against this complication [8, 19]. In our study, patients received treatment for maternal disease immediately after the diagnosis of the disease. Therefore, women who were already under rheumatologic follow-up prior to pregnancy or who had a planned pregnancy were using medications with potential protective effects against congenital heart block before conception. In contrast, pregnant women who were diagnosed during pregnancy initiated treatment at that time. It is important to clarify that no treatment aimed at improving CHB (such as corticosteroids or beta-agonists) was instituted after its diagnosis for this purpose. When corticosteroids were prescribed, the indication was exclusively to manage maternal clinical conditions.

Another finding reinforcing the hypothesis that inflammatory activity constitutes a risk factor for CHB was the observation of significantly lower complement levels in mothers whose fetuses were affected (*p* = 0.008 for C4 and *p* = 0.093 for C3) [14].

In contrast, recent advances have shifted the focus toward targeted therapies that directly interfere with the pathogenic mechanism of antibody-mediated fetal injury. Novel agents targeting Fc receptor–mediated antibody transfer, specifically blockade of the neonatal Fc receptor (FcRn), represent a promising emerging strategy. FcRn inhibitors such as rozanolixizumab have demonstrated, in proof-of-concept human studies, the ability to reduce maternal total IgG and anti-Ro/SSA antibody levels by approximately 65% during the critical window of fetal cardiac vulnerability (gestational weeks 14–28), thereby decreasing placental transfer of pathogenic antibodies [20–23]. Notably, a recent case report described successful prevention of recurrent cardiac neonatal lupus with weekly subcutaneous rozanolixizumab (560 mg), resulting in normal fetal cardiac outcomes, supporting the rationale for further evaluation of FcRn blockade in prospective multicenter trials [20–23].

Other potential confounders identified include the higher prevalence of Sjögren’s syndrome in the CHB group (38.5% vs. 14.2%; *p* = 0.021), the presence of positive ANA (*p* = 0.047), and the significant association between cutaneous manifestations and CHB (*p* < 0.001). It is noteworthy that Sjögren’s syndrome is classically associated with anti-Ro and/or anti-La positivity, which are well-established risk factors for the development of CHB. Similarly, cutaneous manifestations are frequent in patients with systemic lupus erythematosus (SLE), as is ANA positivity, a mandatory classification criterion for SLE [14]. One possible explanation for the higher rate of CHB among pregnant women without SLE is that, in our center, once a diagnosis of congenital heart block is made, anti-Ro, anti-La, and ANA testing is routinely requested for these patients in order to guide counseling and management of future pregnancies.

In our study, the higher cesarean delivery rate among fetuses with CHB may be explained by the increased complexity of fetal well-being assessment in this group. In such cases, the diagnosis of acute fetal distress is more challenging, as cardiotocography is not a reliable tool and Doppler velocimetry must be interpreted with caution. Given these limitations, for patients who did not enter spontaneous labor and did not present in the expulsive phase before 37 weeks of gestation, elective cesarean delivery was indicated between 37 and 38 weeks. These patients with CHB underwent weekly ultrasonographic evaluation of the fetal biophysical profile without cardiotocography from the time of diagnosis until 37 weeks of gestation, when delivery was indicated.

Finally, this study underscores the importance of prenatal diagnosis of fetal cardiac involvement, which enables delivery planning in tertiary referral centers capable of providing advanced neonatal support, including early pacemaker implantation before hospital discharge—a measure required in more than half of the evaluated cases [24]. The main complications following pacemaker implantation for the treatment of neonatal complete atrioventricular block include infection of the implantation site or pacing system, lead dysfunction or failure, lead dislodgement, hematoma, pneumothorax, cardiac tamponade, and complications related to venous or surgical access [19, 24]. Possible explanations for the high infection rate observed in this study include factors related to patient characteristics, procedural complexity, the presence of invasive devices, frequent use of antimicrobial agents and bacterial resistance.

To date, all newborns with CHB from this paper have shown a good response and clinical course following pacemaker implantation. Those newborns who did not undergo pacemaker implantation during the immediate postpartum hospitalization were all submitted to the procedure within the first year of life.

## Conclusion

This study demonstrated a significant association between the presence of maternal anti-Ro and anti-La autoantibodies and the occurrence of congenital heart block (CHB) in newborns. Higher titers of these autoantibodies were also associated with a proportionally increased risk of CHB development. Furthermore, the findings suggest that hydroxychloroquine at a dose of 400 mg daily use may exert a protective effect against the occurrence of fetal heart block, provided that treatment is initiated prior to conception; however, further studies are warranted to confirm this potential benefit since it is also important to acknowledge that one of the main limitations of this study was the small number of participants with congenital heart block, as this is a rare condition.

In light of these findings, it is strongly recommended that pregnancies with positive maternal autoantibodies be managed in tertiary referral centers specialized in high-risk obstetrics, as in severe cases, immediate neonatal intervention—such as pacemaker implantation within the first hours of life—may be required.

## Data Availability

No datasets were generated or analysed during the current study.
